# Evaluation of JC9450 and Neutral Electrolyzed Water in Controlling *Listeria monocytogenes* on Fresh Apples and Preventing Cross-Contamination

**DOI:** 10.3389/fmicb.2019.03128

**Published:** 2020-01-14

**Authors:** Lina Sheng, Xiaoye Shen, Oscar Ulloa, Trevor V. Suslow, Ines Hanrahan, Mei-Jun Zhu

**Affiliations:** ^1^School of Food Science, Washington State University, Pullman, WA, United States; ^2^Department of Plant Sciences, University of California, Davis, Davis, CA, United States; ^3^Washington Tree Fruit Research Commission, Wenatchee, WA, United States

**Keywords:** fresh apple, *Listeria monocytogenes*, neutral electrolyzed water, JC9450, antimicrobial, cross-contamination

## Abstract

Recent multistate outbreaks and recalls of fresh apples due to *Listeria monocytogenes* contamination have increased consumer concerns regarding fresh and processed apple safety. This study aimed to evaluate the antimicrobial efficacy of two sanitizers, mineral oxychloride (JC9450) and neutral electrolyzed water (NEW), for inactivation of *L. monocytogenes* on fresh apples. A 2-min treatment of 0.125% (v/v) JC9450 with 100 ppm free available chlorine (FAC) or NEW with 110 ppm FAC caused 0.9–1.2 log_10_ CFU/apple reduction of *L. monocytogenes* on both Granny Smith and Fuji apples 24 h post-inoculation. Increasing JC9450 concentration to 0.25 and 0.50% significantly improved its bactericidal effect and reduced *L. monocytogenes* on Granny Smith apples by ~2.0 and 3.8 log_10_ CFU/apple, respectively, after a contact time of 2 min. At a shorter contact time of 30 sec, the inactivation efficacy of chlorine and 0.25–0.50% JC9450 against *L. monocytogenes* on apples was significantly reduced compared with the respective 2-min wash. Furthermore, no *L. monocytogenes* was recovered in deionized water prepared antimicrobial wash solution or on non-inoculated apples post-NEW with 110 ppm FAC or 0.125–0.5% JC9450 washes, indicating their ability to prevent cross-contamination. In addition, a 2-min exposure to NEW with 110 ppm FAC and 0.50% JC9450 reduced apple native microbiota including total plate count by 0.14 and 0.65 log_10_ CFU/apple, respectively, and yeast and mold counts by 0.55 and 1.63 log_10_ CFU/apple, respectively. In summary, *L. monocytogenes* attached on apples was difficult to eliminate. JC9450 and NEW demonstrated a dose-dependent reduction in *L. monocytogenes* on apples and successfully prevented cross-contamination, indicating their application potential in post-harvest washes of apples.

## Introduction

*Listeria monocytogenes* is a major foodborne pathogen, which causes more than 1,500 illnesses annually in the United States with a high mortality rate of ~16% (Scallan et al., 2011). Multistate outbreaks attributed to contamination of *L. monocytogenes* on caramel apples (CDC, 2015) and recalls of fresh apples (FDA, 2016, 2017b) and sliced apples (CFIA, 2015) due to potential *L. monocytogenes* contamination highlight the importance of preventive control programs directed at *L. monocytogenes* on fresh apples*. L. monocytogenes* population remained stable on fresh apple surface during 3 months of cold storage (Sheng et al., 2017).

Antimicrobial sanitizer interventions have been widely used for the processing and packing of fresh produce to minimize the risk of foodborne pathogen and prevent the cross-contamination. Chlorine, typically in the form of sodium/calcium hypochlorite, is the most commonly used antimicrobial in the fresh produce industry (Banach et al., 2015). The major antimicrobial components in chlorine solutions are hypochlorous acid (HOCl) and hypochlorite ion (OCl^−^), namely free available chlorine (FAC), where HOCl has a stronger oxidation capacity and antimicrobial efficacy than OCl^−^ (Fukuzaki, 2006; Rahman et al., 2016). The efficacy of chlorine is related to the FAC level, which varies depending on the washing conditions such as pH and organic matter (Francis et al., 2012). Chlorine at commonly used concentration, 50–200 ppm (Suslow, 2005), has a limited efficacy against *L. monocytogenes* on fresh produce (Prado-Silva et al., 2015). A 1-min spray of sodium hypochlorite solution with 200 ppm FAC reduced *L. monocytogenes* inoculated on whole Red Delicious apples at ~1.7 log_10_ CFU/cm^2^ by ~0.9 log_10_ CFU/cm^2^ (Beuchat et al., 1998). Dipping in 100 ppm chlorine for 2 min reduced *L. monocytogenes* inoculated on Granny Smith apples at ~6.4 log_10_ CFU/apple by 0.7–0.9 log_10_ CFU/apple (Shen et al., 2019). There is also safety concern about the production of carcinogenic halogenated by-products resulted from chlorinated organic compounds (Brown et al., 2011; Gil et al., 2016). As a result, chemical and fresh produce industries have been seeking for alternative antimicrobial sanitizers with an improved efficacy and convenience.

JC9450 is a novel sanitizer certified by the National Sanitation Foundation (NSF) and approved for use in potable water systems as a disinfectant (NSF, 2012). The active antimicrobial ingredient of JC9450 is mineral oxychloride or metal oxychloride (MO_x_Cl_y_), which is a chemical compound where oxygen (O) and chlorine (Cl) atoms are bonded to a metal (M) (Yu et al., 2017). Unlike the well-known fungicide copper oxychloride [Cu_4_(OH)_6_Cl_2_], inhibitory effect of which relies on the antifungal efficacy of copper (Cioffi et al., 2005; Peter, 2016), the metals in mineral oxychloride act as catalysts to generate reactive oxygen species (ROS) especially hydroxyl radicals through interaction with water (Yang et al., 2013; Hayyan et al., 2016; Li and Zhang, 2016; Jenfitch, 2018). JC9450 is reported to have a superior antimicrobial efficacy than chlorine; 0.0002% JC9450 and chlorine reduced *Salmonella enterica* ATCC 10708 in liquid broth by ~6.7 and 0.1 log_10_ CFU/ml, respectively (DisinfectWater, 2017). The concentration of mineral oxychloride that inhibited 99% of germination (EC_99_) of conidia of *Penicillium digitatum* was 0.003%, while the EC_99_ of chlorine was 0.01% (Smilanick et al., 2014). However, there is no information available regarding its efficacy against *L. monocytogenes* on fresh apples or in water.

Neutral electrolyzed water (NEW) is produced from the electrochemical reaction of water and salt, and on-site generation creates a sodium-free solution of hypochlorous acid (HOCl) with a high oxidation-reduction potential (Rahman et al., 2016). Over the years, NEW has been evaluated against foodborne pathogens and spoilage microorganisms on various fresh and fresh-cut produce. A 5-min wash with NEW containing 100 ppm FAC (pH ~8.4) reduced *L. innocua* on apple slices by ~0.9 log_10_ CFU/g (Graca et al., 2011). Using NEW wash with 89 ppm FAC (pH ~8.0), there was more than 4.0 log_10_ CFU/cm^2^ reductions of *L. monocytogenes*, *Salmonella*, and *E. coli* O157:H7 inoculated on tomato surface within 1 min (Deza et al., 2003). NEW with 50 ppm FAC (pH ~7.5) reduced *Alicyclobacillus acidoterrestris* spores on apples by ~2.0 log_10_ CFU/apple after 1-min exposure (Torlak, 2014). However, its efficacy against *L. monocytogenes* on fresh apples or in washing solution has not been evaluated. Therefore, the objectives of this study were to evaluate the efficacy of JC9450 and NEW to control *L. monocytogenes* on fresh apples and to prevent cross-contamination.

## Materials and Methods

### *Listeria monocytogenes* Strains and Culture Preparation

*L. monocytogenes* NRRL B-57618 (1/2a, human clinical isolate), NRRL B-33053 (4b, Coleslaw outbreak isolate), and NRRL-33466 (1/2b, environmental isolate) were obtained from the USDA-ARS culture collection [National Center for Agricultural Utilization Research (NRRL), Peoria, IL, USA] and maintained at −80°C in trypticase soy broth [Becton, Dickinson and Company (BD), Sparks, MD, USA] supplemented with 0.6% yeast extract (TSBYE; Fisher Scientific, Fair Lawn, NJ, USA) and 20% glycerol. Each frozen stock culture was reconstituted in TSBYE at 37°C for 24 h. For test cultures, these were sub-cultured into fresh TSBYE and incubated as before. All the experiments were conducted in a biosafety level 2 lab.

### Inoculum Preparation

Following incubation, the cultures were centrifuged at 8,000 × *g* for 5 min at 4°C, and the resulting pellets were washed once and re-suspended in phosphate buffered saline (PBS, pH 7.4). Equal population of each washed *L. monocytogenes* strain (~5 × 10^8^ CFU/ml per strain) was combined to make a three-strain cocktail and diluted to achieve ~10^6^ CFU/ml for apple inoculation. The high contamination level was chosen to enable accurate detection of bacterial count reductions.

### Inoculation of Apples

Different lots of unwaxed Granny Smith apples (GSA) and Fuji apples of commercial maturity, the optimum maturity for either the fresh market or the storage (Girschik et al., 2017), were donated by Allan Brothers Inc. (Naches, WA, USA) and Stemilt Growers LLC (Wenatchee, WA, USA) and stored at 4°C. Apples of uniform size (~200 g/apple) with stems left fully intact and devoid of cuts, bruises, or scars were selected for the experiment. The whole apples were rinsed with cold tap water, dried and then dip-inoculated in the *Listeria* cocktail (~10^6^ CFU/ml) at room temperature (RT, ~22.5°C) as previously described (Sheng et al., 2017), and dried at RT and ambient environmental relative humidity (RH) for 24 or 48 h. Twelve apples were randomly sampled immediately following inoculation (0 h) and at 24 and 48 h post-inoculation for enumeration following the procedure described in section “*Listeria monocytogenes* Enumeration” to confirm the persistent population density of inoculated *L. monocytogenes*.

### Antimicrobial Intervention on Apple Surfaces

JC9450 was kindly provided by Jenfitch LLC (Walnut Creek, CA, USA). JC9450 consists of 6.7–9.5% mineral oxychloride (CAS#1332-17-8), 0.02–0.67% sodium hydroxide, and water. JC9450 was tested at 0.125, 0.25, and 0.50% (v/v). NEW (Disinfectant 275) was donated by AquaOx (Loxahatchee, FL, USA) and tested at 1:15, 1:7, and 1:3 dilutions corresponding to 22.5, 55, 110 ppm FAC, respectively. Chlorine, prepared from Accu-Tab (Calcium hypochlorite, Pace International, Wapato, WA, USA) with ~100 ppm FAC, was used as control. All solutions were prepared with deionized water and applied right after preparation. The pH and oxidation/reduction potential (ORP) of wash solutions were measured at RT with Orion 8302BNUMD ROSS Ultra pH/ATC Triode (Thermo Scientific, Waltham, WA, USA) and Orion 9678BNWP electrode (Thermo Scientific), respectively, connected to an Orion Versa Star Pro advanced electrochemistry meter (Thermo Scientific). FAC was measured with a Taylor K-2006 complete test kit (Taylor Technologies, Sparks, MD, USA), and there was no difference in FAC in tested solutions before and after wash. The pH, ORP, and FAC content for all wash solutions are listed in Table 1.

**Table 1:** Physicochemical properties of antimicrobials used in this study.

Treatment	pH	ORP (mV)	FAC (ppm)
Water	6.65 ± 0.15	346.1 ± 12.7	0.0 ± 0.0
Chlorine	6.82 ± 0.01^*^	882.5 ± 6.3	113.3 ± 1.0
0.125% JC9450	6.81 ± 0.00^*^	854.3 ± 4.7	100.0 ± 0.0
0.25% JC9450	6.81 ± 0.01^*^	887.5 ± 4.4	200.0 ± 0.0
0.50% JC9450	6.82 ± 0.00^*^	908.8 ± 1.8	400.0 ± 0.0
1:3 NEW	6.88 ± 0.15	883.7 ± 8.9	110 ± 0.0
1:7 NEW	6.74 ± 0.07	870.0 ± 8.0	55.0 ± 0.0
1:15 NEW	6.58 ± 0.02	823.2 ± 7.2	22.5 ± 0.0

*JC9450, mineral oxychloride; NEW, neutral electrolyzed water; ORP, oxidation reduction potential; FAC, free available chlorine; mean ± SEM, n = 9*.

* *pH of solution was adjusted to 6.8 with 6 N HCl. All measurements were conducted at room temperature (~22.5°C)*.

For each sanitizer and contact time treatment combination, a set of 12 apples was submerged in 3 L of respective antimicrobial solution at RT and agitated manually for 2 min or 30 sec. The deionized water wash was used as a negative control. After treatment, the apples were removed immediately from the sanitizing solution and analyzed to estimate viable *L. monocytogenes* populations. Each sanitizer and time treatment combination was repeated independently three times.

### Evaluation of Cross-Contamination

To evaluate the efficacy of the aforementioned antimicrobial sanitizers in preventing cross-contamination of *L. monocytogenes* during apple washing, inoculated apples were introduced along with non-inoculated apples at either 1:10 or 6:6 ratio to respective sanitizer solution (Pao et al., 2007; Nou and Luo, 2010). They were treated as described in section “Antimicrobial Intervention on Apple Surfaces”. Each treatment was repeated independently three times.

### *Listeria monocytogenes* Enumeration

To enumerate survival of *L. monocytogenes* on inoculated apples, each apple was placed into a stomacher bag with 10 ml sterile PBS and hand-rubbed for 1.5 min to detach adherent cells from apple surfaces (Sheng et al., 2018). Rub solutions were 10-fold serially diluted with sterile PBS, and 0.1 or 1 ml (333 μl/plate, 3 plates) from appropriate dilutions was plated on duplicate TSAYE (TSBYE with 1.5% agar) plates and then overlaid with modified Oxford agar (MOX, BD) to differentiate *L. monocytogenes* from indigenous apple microbiota (Kang and Fung, 1999).

Residual populations of *L. monocytogenes* in the wash water were enumerated by serially diluting and then plating following the above quantitative method. Residual *L. monocytogenes* in the spent antimicrobial solutions was enumerated by filtration method. Briefly, 100 ml wash solution was filtrated through a disposable 0.45 μm analytical test filter funnel (Thermo Scientific), rinsed twice with sterile PBS, and placed on TSAYE and CHROMagar™ *Listeria* (DRG International Inc. Springfield, NJ, USA) plates followed by incubation at 37°C for 48 h. Presumptive colonies were further confirmed by PCR targeting invasion-associated secreted endopeptidase (*iap*) gene (FDA, 2017a).

To examine the potential cross-contamination of *L. monocytogenes*, non-inoculated apples were hand rubbed in the same manner as inoculated apples, and 1 ml of rub solution was transferred into 9 ml buffered *Listeria* enrichment broth (BLEB, BD), incubated at 30°C for 48 h, and streaked onto CHROMagar™ *Listeria* plates (FDA, 2017a). The plates were incubated at 37°C for 48 h to determine the presence or absence of *L. monocytogenes.* Presumptive colonies were confirmed by PCR as mentioned above.

### Background Microbiota Enumeration

Non-inoculated apples post-sanitizer treatments were rubbed for 1.5 min to detach the resident microbiota on apple surface. The rub solutions were serially diluted and then plated onto TSAYE for total plate count (TPC) and potato dextrose agar (PDA, BD) plates for yeast and mold (Y/M) counts, respectively. TSAYE plates were incubated at 35 ± 1°C for 48 h, while PDA plates were incubated at RT for 3~5 days.

### Statistical Analysis

Data were analyzed by GLM from Statistical Analysis Systems (SAS, Cary, NC, USA). Mean values were compared by least significant difference (LSD) multiple-comparison test. *p* values of less than 0.05 were considered statistically significant. Each experiment was repeated three times independently. Results were reported as mean ± standard error mean (SEM). Log reduction of *L. monocytogenes* or background microbiota from apples was averaged from three independent experiments with 12 apples/treatment in each independent study, *n* = 36. Three spent water samples were collected in each independent cross-contamination study, hence a total of nine samples per treatment.

## Results

### Antimicrobial Efficacy of JC9450 and Neutral Electrolyzed Water

**Figure 1:**
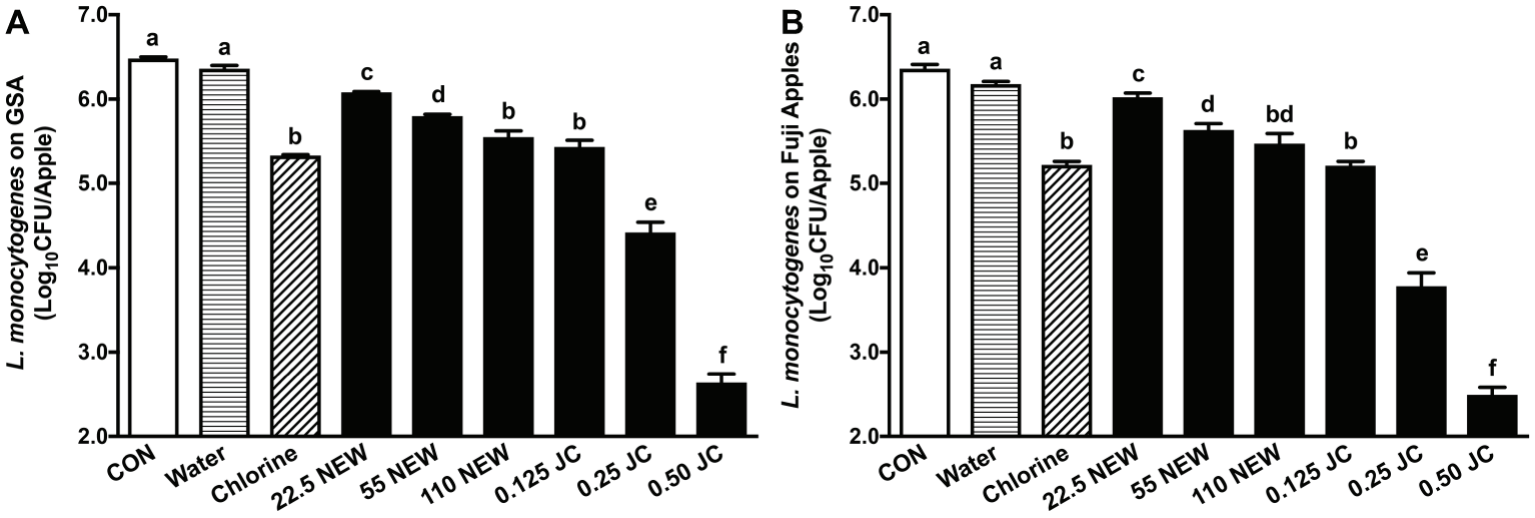
Efficacy of JC9450 and neutral electrolyzed water against *Listeria monocytogenes* on fresh apples 24 h post-inoculation with a contact time of 2 min. **(A)** *L. monocytogenes* count on Granny Smith apples (GSA) post-sanitizer treatments; **(B)** *L. monocytogenes* count on Fuji apples post-sanitizer treatments. Mean ± SEM, averaged from three independent studies with 12 apples/treatment in each independent study (*n* = 36). CON, untreated control; JC, JC9450, mineral oxychloride, %; NEW, neutral electrolyzed water, tested at 22.5, 55, and 110 ppm free available chlorine. Chlorine at ~110 ppm free available chlorine was used as a positive control. Histogram bars without common letter differ significantly (*p* < 0.05).

Antimicrobial efficacy of JC9450 and NEW was first assessed and compared against *L. monocytogenes* on GSA, the variety associated with a recent listeriosis caramel apple outbreak (CDC, 2015), and Fuji apple, which is a commercially important variety and has a different surface structure than GSA (Hall, 1966). The initial *L. monocytogenes* inoculation level on GSA and Fuji apples was 6.24 ± 0.06 and 6.18 ± 0.03 log_10_ CFU/apple, respectively. After 24 h at RT, the populations of *L. monocytogenes* on GSA and Fuji apples were 6.48 ± 0.07 and 6.36 ± 0.05 log_10_ CFU/apple, respectively (Figure 1). NEW with 110 ppm FAC and 0.125% JC9450 reduced *L. monocytogenes* on GSA by ~1.0 log_10_ CFU/apple (Figure 1A). Increasing JC9450 concentration improved its efficacy; 0.25 and 0.50% of JC9450 caused ~2.1 and 3.8 log_10_ CFU/apple reductions of *L. monocytogenes* on GSA, respectively (Figure 1A). Reducing NEW concentrations to 55 and 22.5 ppm FAC significantly decrease its efficacy against *L. monocytogenes* on fresh apples. *L. monocytogenes* on Fuji apples exhibited the same degree of resistance (*p* < 0.05) in response to respective antimicrobial treatment as GSA (Figure 1B). Therefore, only GSA was used in subsequent experiments.

### Influence of *Listeria monocytogenes* Attachment Time and Sanitizer Contact Time on Antimicrobial Efficacy of JC9450 and Neutral Electrolyzed Water

Bacterial attachment time before sanitizer intervention is reported to impact antimicrobial efficacy of sanitizers (Lang et al., 2004). Therefore, the antimicrobial efficacy of selected sanitizers was tested against *L. monocytogenes* on apples at 24 and 48 h post-inoculation. The counts of *L. monocytogenes* on apples 24 and 48 h post-inoculation were 6.48 ± 0.02 and 6.45 ± 0.02 log_10_ CFU/apple, respectively. At 24 h post-inoculation, reducing contact time from 2 min to 30 sec significantly decreased the antimicrobial efficacy of chlorine and 0.25–0.50% JC9450 (Table 2). At 48 h post-inoculation, shortening contact time to 30 sec had no influence on the inhibitory effect of all the tested sanitizers except 0.50% JC9450 (Table 2).

**Table 2:** Influence of *Listeria monocytogenes* attachment time and sanitizer contact time on the antimicrobial efficacy of neutral electrolyzed water and JC9450 against *Listeria monocytogenes* on fresh apples.

Treatment	24 h post-inoculation		48 h post-inoculation	
	30-sec exposure	2-min exposure	30-sec exposure	2-min exposure
Water	0.11 ± 0.01^aA^	0.12 ± 0.02^aA^	0.09 ± 0.03^aA^	0.09 ± 0.02^aA^
Chlorine	0.90 ± 0.04^bA^	1.15 ± 0.01^bB^	0.88 ± 0.07^bA^	1.05 ± 0.10^bA^
NEW	0.75 ± 0.09^bA^	0.93 ± 0.09^bA^	0.70 ± 0.10^bA^	0.88 ± 0.08^bA^
0.125% JC9450	0.91 ± 0.04^bA^	1.04 ± 0.08^bA^	0.82 ± 0.04^bA^	0.88 ± 0.10^bA^
0.25% JC9450	1.58 ± 0.05^cA^	2.06 ± 0.12^cB^	1.56 ± 0.03^cA^	1.84 ± 0.10^cA^
0.50% JC9450	3.27 ± 0.12^dA^	3.84 ± 0.10^dB^	3.09 ± 0.14^dA^	3.61 ± 0.05^dB^

*Results were expressed in count reduction, log_10_ CFU/apple. At both 24 and 48 h post-inoculation, the count of L. monocytogenes was ~6.5 log_10_ CFU/apple. JC9450, mineral oxychloride; NEW, neutral electrolyzed water, 110 ppm free available chlorine. Chlorine at ~110 ppm free available chlorine was used as a positive control*.

*Superscript letters ^(a–d)^ mean within a column without common letter differ significantly (p < 0.05)*.

*Superscript letters ^(A,B)^ mean across rows within each post-inoculation time without common letters differ significantly (p < 0.05). Mean ± SEM, n = 36. Each treatment was repeated independently three times with 12 apples per treatment per independent study*.

### Efficacy of JC9450 and Neutral Electrolyzed Water in Prevention of Cross-Contamination

A 2-min wash with water alone transferred ~4.6 log_10_ CFU/ml *L. monocytogenes* from inoculated apples to wash solution, while residual *L. monocytogenes* in wash solutions of chlorine, NEW with 110 ppm FAC, and 0.125–0.50% JC9450 were reduced to under the detectable level (1 CFU/100 ml) at contact times of 30 sec–2 min (data not shown). No *L. monocytogenes* was detected on non-inoculated apples following 2-min NEW (110 ppm FAC) or JC9450 (0.125–0.50%) washes regardless of the contamination level (Table 3). However, the 2-min water wash without any antimicrobial transferred 3.8–4.2 log_10_ CFU/apple of *L. monocytogenes* to non-inoculated apples (Table 3). These data, collectively, indicated that JC9450 and NEW have the potential to prevent cross-contamination from both fruit-to-water and fruit-to-fruit.

**Table 3:** Efficacy of neutral electrolyzed water and JC9450 for the prevention of cross-contamination of *Listeria monocytogenes* among apples.

Inoculated: uninoculated	Treatment	Inoculated apple (log_10_ CFU/apple)	Non-inoculated apple (log_10_ CFU/apple) (positive^*^/total apple)
1:10	Water	6.40 ± 0.01^a^	3.79 ± 0.07 (10/10)
	Chlorine	5.48 ± 0.07^b^	ND (0/10)
	NEW	5.46 ± 0.09^b^	ND (0/10)
	0.125% JC9450	5.42 ± 0.11^b^	ND (0/10)
	0.25% JC9450	4.19 ± 0.11^c^	ND (0/10)
	0.50% JC9450	2.76 ± 0.10^d^	ND (0/10)
6:6	Water	6.47 ± 0.05^a^	4.15 ± 0.11 (6/6)
	Chlorine	5.60 ± 0.03^b^	ND (0/6)
	NEW	5.61 ± 0.04^b^	ND (0/6)
	0.125% JC9450	5.58 ± 0.07^b^	ND (0/6)
	0.25% JC9450	4.27 ± 0.02^c^	ND (0/6)
	0.50% JC9450	2.86 ± 0.08^d^	ND (0/6)

* *L. monocytogenes positive apple meant presence of L. monocytogenes on CHROMagar Listeria plates after enrichment of the detached microbial suspension. Apples were inoculated with ~6.5 log_10_ CFU/apple of L. monocytogenes. JC9450, mineral oxychloride; NEW, neutral electrolyzed water, 110 ppm free available chlorine; ND, not detected after enrichment. Chlorine at ~110 ppm free available chlorine was used as a positive control*.

*Superscript letters ^(a–d)^ mean within a column without common letter differ significantly (p < 0.05). Mean ± SEM. Each treatment was repeated independently three times with 11–12 apples per treatment per independent study*.

### Bactericidal Effects of JC9450 and Neutral Electrolyzed Water Against Apple Background Microbiota

**Figure 2:**
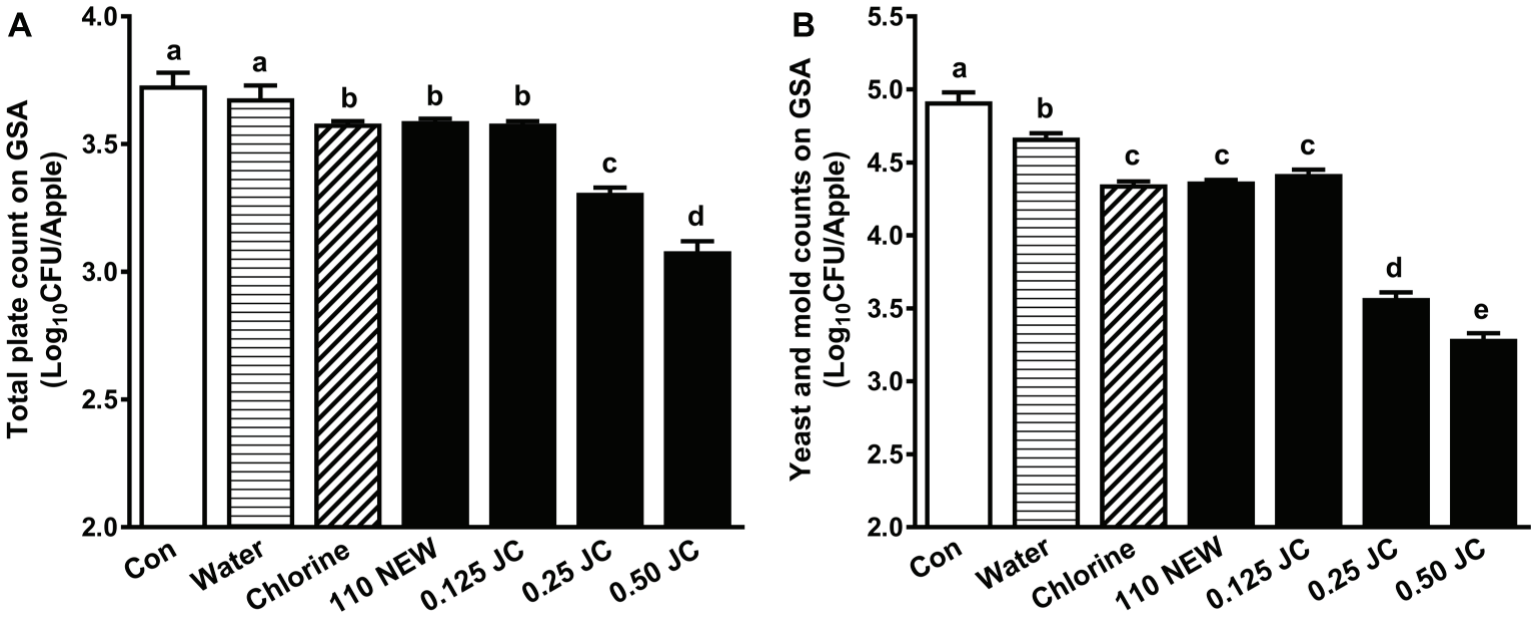
Effects of JC9450 and neutral electrolyzed water against background microbiota on Granny Smith apples (GSA). **(A)** Total plate counts (TPC) of resident bacteria on GSA post-sanitizer treatments; **(B)** yeast and mold (Y/M) counts on GSA post-sanitizer treatments; mean ± SEM, averaged from three independent studies with 12 apples/treatment in each independent study (*n* = 36). CON, untreated control; JC, JC9450, mineral oxychloride, %; NEW, neutral electrolyzed water, 110 ppm free available chlorine. Chlorine at ~110 ppm free available chlorine was used as a positive control. Histogram bars without common letter differ significantly (*p* < 0.05).

Both NEW at 110 ppm FAC and 0.125% JC9450 along with chlorine have a similar but limited efficacy against naturally presented background microbiota; they caused 0.14–0.15 and 0.50–0.60 log_10_ CFU/apple reduction of TPC and Y/M, respectively (Figure 2). Increasing JC9450 concentrations improved its efficacy; a 2-min exposure to 0.50% JC9450 reduced TPC and Y/M counts by 0.65 and 1.63 log_10_ CFU/apple, respectively (Figure 2).

## Discussion

### Effect of Neutral Electrolyzed Water and JC9450 Against *Listeria monocytogenes* on Fresh Apple

NEW at 110 ppm FAC and 0.125% JC9450 showed a comparable but limited antimicrobial efficacy against *L. monocytogenes* on apples as did conventional chlorine treatment. Similarly, spray application of sodium hypochlorite solution with 200 ppm FAC reduced *L. monocytogenes* inoculated on whole Red Delicious apples by ~0.9 log_10_ CFU/cm^2^ after a contact time of 1 min (Beuchat et al., 1998). A 1-min exposure to NEW at 89 ppm FAC reduced *L. innocua*, a non-pathogenic species closely related to *L. monocytogenes*, in lettuce by ~1.2 log_10_ CFU/g (Abadias et al., 2008). Increasing JC9450 concentration to 0.50% enhanced its antimicrobial efficacy, possibly due to the increased production of bactericidal ROS.

### Factors Influencing the Antimicrobial Efficacy of JC9450 and Neutral Electrolyzed Water

The morphology of surface wax, where pathogens attach on intact apple surface (Burnett et al., 2000), varies among different apple varieties (Hall, 1966). These surface morphologies might influence the interaction between bacteria and apple surface and subsequent inactivation. However, our results suggested that the JC9450 and NEW had similar efficacies against *L. monocytogenes* on GSA and Fuji apples. In support of our findings, a 30-sec exposure to 200 ppm chlorine reduced *Penicillium expansum* by 1.3–1.6 log_10_ spores/g on Red Delicious, Gala, and Fuji apples (Salomao et al., 2008). The survival of *L. monocytogenes* on GSA and Fuji apples was similar during a 3-month cold storage (Sheng et al., 2017).

Reducing contact times significantly reduced the efficacy of chlorine and 0.25–0.50% JC9450 on freshly contaminated apples (24 h post-inoculation). Similarly, a 25 ppm HOCl wash for 15 sec and 2 min reduced *Salmonella* 2 h post-inoculated on tomatoes by ~2.4 and 6.0 log_10_ CFU/tomato, respectively (Gereffi et al., 2015). By contrast, a 200 ppm chlorine wash for both 3 and 10 min reduced *L. monocytogenes* on cantaloupe surface 2 h post-inoculation by ~1.0 log_10_ CFU/cm^2^ (Upadhyay et al., 2014). Chlorine wash with 100 ppm FAC for 1 and 3 min reduced *E. coli* O157:H7 2 h post-inoculated onto Red Delicious apples by ~4.6 log_10_ CFU/apple (Baskaran et al., 2013).

The impact of contact time was lost for *L. monocytogenes* on apples after prolonged drying, indicating an increased antimicrobial tolerance of *L. monocytogenes* on fresh apples to JC9450 and NEW. Similarly, antimicrobial efficacy of 100 ppm chlorine against *L. monocytogenes* on lettuce 24 h post-inoculation was reduced compared to that at 6 h post-inoculation, which resulted in ~2.2 and 3.5 log_10_ CFU/g reduction after a 2-min exposure, respectively (Olmez and Temur, 2010). Chlorine at 200 ppm with an exposure time of 5 min caused ~3.8 and 1.8 log_10_ CFU/tomato reductions in *E. coli* O157:H7 post-1 h and -24 h inoculation, respectively (Lang et al., 2004). A 2-min exposure to 80 ppm PAA reduced *L. monocytogenes* on GSA by ~2.2 and 1.7 log_10_ CFU/apple after 24 and 48 h of attachment, respectively (Shen et al., 2019). The increased resistance with prolonged attachment time might be due to the increased binding strength of bacteria to produce surface and/or maturation of cell aggregates and protective extracellular materials (Elhariry, 2011). Lengthened attachment time might also induce bacterial desiccation stress response, which may cross-protect the bacterial cells against sanitizers (Lou and Yousef, 1997). Biofilm formation and development of cell aggregates during extended adhesion could further contribute to the increased antimicrobial resistance (Olmez and Temur, 2010). However, *Salmonella* attached on whole cantaloupe rind for 24 and 72 h demonstrated a similar resistance to 200 ppm chlorine, as its levels were reduced by ~2.5 log_10_ CFU/cm^2^ after a contact time of 2 min (Ukuku and Fett, 2006).

### Assessment of the Efficacy of JC9450 and Neutral Electrolyzed Water in Preventing Cross-Contamination

During washing, pathogens in contaminated fresh produce can be directly transferred to wash water, which further transfers pathogens to contaminant-free produce (Allende et al., 2008). In this study, a single apple harboring *L. monocytogenes* was able to contaminate a whole batch of 10 clean apples with ~3.8 log_10_ CFU/apple in the absence of sanitizer. Similarly, a 2-min water wash transferred ~5.0 log_10_ CFU/tomato of *Salmonella* from inoculated tomato (8.3 log_10_ CFU/tomato) to non-inoculated tomatoes (Gereffi et al., 2015). These data indicated that during post-harvest processing, a single contaminated fresh produce has the potential to compromise the whole batch or entire lot of fresh produce. It is of great necessity that the antimicrobial used in fresh produce wash can prevent cross-contamination. NEW and JC9450 at ~100 ppm FAC eliminated residual *L. monocytogenes* in the spent wash solutions and prevented cross-contamination to clean apples at both low and high contamination levels. In support of our data, NEW with 89 ppm FAC wash for 30 sec or 1 min eliminated *L. monocytogenes* in the spent solution post-*L. monocytogenes*-inoculated tomato washing (Deza et al., 2003).

### Efficacy of JC9450 and Neutral Electrolyzed Water to Reduce Apple Background Microbiota

Washes with acidic electrolyzed water (pH ~2.5) at 50 ppm FAC have been reported to reduce TPC and Y/M on cabbage and shredded carrots by ~2.0 log_10_ CFU/g after a 3-min exposure (Rahman et al., 2010, 2011). NEW at 50 ppm FAC and 3-min exposure reduced TPC on fresh-cut carrot slices by 1.8 log_10_ CFU/g (Izumi, 1999). In this study, however, JC9450 or NEW at 100 FAC had a limited efficacy in removing background bacteria and Y/M. A similar phenomenon was observed where a 30 sec treatment with 2% sodium orthophenylphenate formulated detergent did not decrease background bacteria of citrus fruit (Pao and Brown, 1998). The different efficacy against background microbiota might be due to the different produce surface composition and structure as well as indigenous bacterial species and ecology.

## Conclusion

JC9450 and NEW at ~100 ppm FAC successfully prevented *L. monocytogenes* cross-contamination from both fruit-to-water and fruit-to-fruit but showed similar yet limited efficacy against *L. monocytogenes* on fresh apples. Future research is needed to evaluate the performance of both sanitizers in water with organic matter to mimic the commercial conditions. Meanwhile, the apple industry should continue to implement good manufacturing practices and preventive controls to minimize the contamination of products with *L. monocytogenes* or other pathogens, through a system-based framework and approach.

## Data Availability Statement

All datasets generated for this study are included in the article.

## Author Contributions

LS performed the experiment and wrote the manuscript. XS and OU assisted the sample analyses. M-JZ and LS designed the experiment. M-JZ, IH, and TS revised the manuscript.

### Conflict of Interest

The authors declare that the research was conducted in the absence of any commercial or financial relationships that could be construed as a potential conflict of interest.

## References

[ref1] Abadias M. Usall J. Oliveira M. Alegre I. Vinas I. (2008). Efficacy of neutral electrolyzed water (NEW) for reducing microbial contamination on minimally-processed vegetables. Int. J. Food Microbiol. 123, 151–158. 10.1016/j.ijfoodmicro.2007.12.008, PMID: 18237810

[ref2] Allende A. Selma M. V. Lopez-Galvez F. Villaescusa R. Gil M. I. (2008). Impact of wash water quality on sensory and microbial quality, including *Escherichia coli* cross-contamination, of fresh-cut escarole. J. Food Prot. 71, 2514–2518. 10.4315/0362-028X-71.12.2514, PMID: 19244906

[ref3] Banach J. L. Sampers I. Van Haute S. van der Fels-Klerx H. J. (2015). Effect of disinfectants on preventing the cross-contamination of pathogens in fresh produce washing water. Int. J. Environ. Res. Public Health 12, 8658–8677. 10.3390/ijerph120808658, PMID: 26213953 PMC4555240

[ref4] Baskaran S. A. Upadhyay A. Kollanoor-Johny A. Upadhyaya I. Mooyottu S. Amalaradjou M. A. R.. (2013). Efficacy of plant-derived antimicrobials as antimicrobial wash treatments for reducing enterohemorrhagic *Escherichia coli* O157:H7 on apples. J. Food Sci. 78, M1399–M1404. 10.1111/1750-3841.12174, PMID: 24024692

[ref5] Beuchat L. R. Nail B. V. Adler B. B. Clavero M. R. S. (1998). Efficacy of spray application of chlorinated water in killing pathogenic bacteria on raw apples, tomatoes, and lettuce. J. Food Prot. 61, 1305–1311. 10.4315/0362-028X-61.10.1305, PMID: 9798146

[ref6] Brown D. Bridgeman J. West J. R. (2011). Predicting chlorine decay and THM formation in water supply systems. Rev. Environ. Sci. Biotechnol. 10, 79–99. 10.1007/s11157-011-9229-8

[ref7] Burnett S. L. Chen J. Beuchat L. R. (2000). Attachment of *Escherichia coli* O157:H7 to the surfaces and internal structures of apples as detected by confocal scanning laser microscopy. Appl. Environ. Microbiol. 66, 4679–4687. 10.1128/AEM.66.11.4679-4687.2000, PMID: 11055910 PMC92366

[ref8] CDC (2015). Multistate outbreak of listeriosis linked to commercially produced, prepackaged caramel apples made from Bidart Bros. apples (final update). Available at: https://www.cdc.gov/listeria/outbreaks/caramel-apples-12-14/index.html (Accessed October 17, 2019).

[ref9] CFIA (2015). Sliced apples and products containing sliced apples recalled due to *Listeria monocytogenes*. Available at: http://www.inspection.gc.ca/about-the-cfia/newsroom/food-recall-warnings/complete-listing/2015-04-30/eng/1430431517170/1430431518530 (Accessed October 17, 2019).

[ref10] Cioffi N. Torsi L. Ditaranto N. Tantillo G. Ghibelli L. Sabbatini L. (2005). Copper nanoparticle/polymer composites with antifungal and bacteriostatic properties. Chem. Mater. 17, 5255–5262. 10.1021/cm0505244

[ref11] Deza M. A. Araujo M. Garrido M. J. (2003). Inactivation of *Escherichia coli* O157:H7, *Salmonella enteritidis* and *Listeria monocytogenes* on the surface of tomatoes by neutral electrolyzed water. Lett. Appl. Microbiol. 37, 482–487. 10.1046/j.1472-765X.2003.01433.x, PMID: 14633103

[ref12] DisinfectWater (2017). JC9400 SERIES Does it fit with EPA’s green chemistry program? Available at: http://disinfectwater.com/wp-content/uploads/2017/08/JC-9450-GREEN-CHEMISTRY-REVIEW.pdf (Accessed October 17, 2019).

[ref13] Elhariry H. M. (2011). Attachment strength and biofilm forming ability of *Bacillus cereus* on green-leafy vegetables: cabbage and lettuce. Food Microbiol. 28, 1266–1274. 10.1016/j.fm.2011.05.004, PMID: 21839375

[ref14] FDA (2016). Fresh from Texas recalls apple product beacuse of possible health risk. Available at: https://www.fda.gov/Food/NewsEvents/ucm494345.htm (Accessed October 17, 2019).

[ref15] FDA (2017a). BAM: detection and enumeration of *Listeria monocytogenes*. Available at: https://www.fda.gov/Food/FoodScienceResearch/LaboratoryMethods/ucm071400.htm (Accessed October 17, 2019).

[ref16] FDA (2017b). Jack Brown Produce, Inc. recalls Gala, Fuji, Honeycrisp and Golden Delicious apples due to possible health risk. Available at: https://www.fda.gov/Safety/Recalls/ucm589722.htm (Accessed October 17, 2019).

[ref17] Francis G. A. Gallone A. Nychas G. J. Sofos J. N. Colelli G. Amodio M. L.. (2012). Factors affecting quality and safety of fresh-cut produce. Crit. Rev. Food Sci. Nutr. 52, 595–610. 10.1080/10408398.2010.503685, PMID: 22530712

[ref18] Fukuzaki S. (2006). Mechanisms of actions of sodium hypochlorite in cleaning and disinfection processes. Biocontrol Sci. 11, 147–157. 10.4265/bio.11.147, PMID: 17190269

[ref19] Gereffi S. Sreedharan A. Schneider K. R. (2015). Control of *Salmonella* cross-contamination between green round tomatoes in a model flume system. J. Food Prot. 78, 1280–1287. 10.4315/0362-028X.JFP-14-524, PMID: 26197278

[ref20] Gil M. I. Marin A. Andujar S. Allende A. (2016). Should chlorate residues be of concern in fresh-cut salads? Food Control 60, 416–421. 10.1016/j.foodcont.2015.08.023

[ref21] Girschik L. Jones J. E. Kerslake F. L. Robertson M. Dambergs R. G. Swarts N. D. (2017). Apple variety and maturity profiling of base ciders using UV spectroscopy. Food Chem. 228, 323–329. 10.1016/j.foodchem.2017.02.012, PMID: 28317730

[ref22] Graca A. Abadias M. Salazar M. Nunes C. (2011). The use of electrolyzed water as a disinfectant for minimally processed apples. Postharvest Biol. Technol. 61, 172–177. 10.1016/j.postharvbio.2011.04.001

[ref23] Hall D. (1966). A study of the surface wax deposits on apple fruit. Aust. J. Biol. Sci. 19, 1017–1026. 10.1071/BI9661017

[ref24] Hayyan M. Hashim M. A. AlNashef I. M. (2016). Superoxide ion: generation and chemical implications. Chem. Rev. 116, 3029–3085. 10.1021/acs.chemrev.5b00407, PMID: 26875845

[ref25] Izumi H. (1999). Electrolyzed water as a disinfectant for fresh-cut vegetables. J. Food Sci. 64, 536–539. 10.1111/j.1365-2621.1999.tb15079.x

[ref26] Jenfitch (2018). Advanced oxidant using mineral oxychloride technology. Available at: https://jenfitch.com/advanced-oxidant-using-mineral-oxychloride-technology-to-help-improve-the-removal-of-pathogens-organic-contaminants-and-inorganic-contaminants/ (Accessed October 17, 2019).

[ref27] Kang D. H. Fung D. Y. C. (1999). Thin agar layer method for recovery of heat-injured *Listeria monocytogenes*. J. Food Prot. 62, 1346–1349. 10.4315/0362-028X-62.11.1346, PMID: 10571328

[ref28] Lang M. M. Harris L. J. Beuchat L. R. (2004). Evaluation of inoculation method and inoculum drying time for their effects on survival and efficiency of recovery of *Escherichia coli* O157:H7, *Salmonella*, and *Listeria monocytogenes* inoculated on the surface of tomatoes. J. Food Prot. 67, 732–741. 10.4315/0362-028X-67.4.732, PMID: 15083725

[ref29] Li J. Zhang L. Z. (2016). Synthesis and facet-dependent properties of layered BiOCl photocatalysts. Cham: Springer.

[ref30] Lou Y. Yousef A. E. (1997). Adaptation to sublethal environmental stresses protects *Listeria monocytogenes* against lethal preservation factors. Appl. Environ. Microbiol. 63, 1252–1255. PMID: 9097420 10.1128/aem.63.4.1252-1255.1997 PMC168417

[ref31] Nou X. Luo Y. (2010). Whole-leaf wash improves chlorine efficacy for microbial reduction and prevents pathogen cross-contamination during fresh-cut lettuce processing. J. Food Sci. 75, M283–M290. 10.1111/j.1750-3841.2010.01630.x, PMID: 20629885

[ref32] NSF (2012). Drinking water treatment chemicals-health effects. Available at: http://info.nsf.org/Certified/PwsChemicals/Listings.asp?Company=C0165759& (Accessed March 04, 2018).

[ref33] Olmez H. Temur S. D. (2010). Effects of different sanitizing treatments on biofilms and attachment of *Escherichia coli* and *Listeria monocytogenes* on green leaf lettuce. LWT-Food Sci. Technol. 43, 964–970. 10.1016/j.lwt.2010.02.005

[ref34] Pao S. Brown G. E. (1998). Reduction of microorganisms on citrus fruit surfaces during packinghouse processing. J. Food Prot. 61, 903–906. 10.4315/0362-028X-61.7.903, PMID: 9678178

[ref35] Pao S. Kelsey D. F. Khalid M. F. Ettinger M. R. (2007). Using aqueous chlorine dioxide to prevent contamination of tomatoes with *Salmonella enterica* and *Erwinia carotovora* during fruit washing. J. Food Prot. 70, 629–634. 10.4315/0362-028X-70.3.629, PMID: 17388051

[ref36] Peter K. A. (2016). Demystifying copper for disease management. Available at: https://agresearch.umd.edu/sites/agresearch.umd.edu/files/_docs/locations/wye/2016%20Winter%20meeting_Copper.pdf (Accessed October 17, 2019).

[ref37] Prado-Silva L. Cadavez V. Gonzales-Barron U. Rezende A. C. B. Sant'Ana A. S. (2015). Meta-analysis of the effects of sanitizing treatments on *Salmonella*, *Escherichia coli* O157:H7, and *Listeria monocytogenes* inactivation in fresh produce. Appl. Environ. Microbiol. 81, 8008–8021. 10.1128/AEM.02216-15, PMID: 26362982 PMC4651082

[ref38] Rahman S. M. E. Jin Y. G. Oh D. H. (2010). Combined effects of alkaline electrolyzed water and citric acid with mild heat to control microorganisms on cabbage. J. Food Sci. 75, M111–M115. 10.1111/j.1750-3841.2009.01507.x, PMID: 20492239

[ref39] Rahman S. M. E. Jin Y. G. Oh D. H. (2011). Combination treatment of alkaline electrolyzed water and citric acid with mild heat to ensure microbial safety, shelf-life and sensory quality of shredded carrots. Food Microbiol. 28, 484–491. 10.1016/j.fm.2010.10.006, PMID: 21356455

[ref40] Rahman S. M. E. Khan I. Oh D. H. (2016). Electrolyzed water as a novel sanitizer in the food industry: current trends and future perspectives. Compr. Rev. Food Sci. Food Saf. 15, 471–490. 10.1111/1541-4337.12200 33401818

[ref41] Salomao B. C. M. Aragao G. M. F. Churey J. J. Worobo R. W. (2008). Efficacy of sanitizing treatments against *Penicillium expansum* inoculated on six varieties of apples. J. Food Prot. 71, 643–647. 10.4315/0362-028X-71.3.643, PMID: 18389716

[ref42] Scallan E. Hoekstra R. M. Angulo F. J. Tauxe R. V. Widdowson M. A. Roy S. L.. (2011). Foodborne illness acquired in the United States-major pathogens. Emerg. Infect. Dis. 17, 7–15. 10.3201/eid1701.P11101, PMID: 21192848 PMC3375761

[ref43] Shen X. Sheng L. Gao H. Hanrahan I. Suslow T. Zhu M. J. (2019). Enhanced efficacy of peroxyacetic acid against *Listeria monocytogenes* on fresh apples at elevated temperature. Front. Microbiol. 10:1196. 10.3389/fmicb.2019.01196 31275249 PMC6591317

[ref44] Sheng L. Edwards K. Tsai H. C. Hanrahan I. Zhu M. J. (2017). Fate of *Listeria monocytogenes* on fresh apples under different storage temperatures. Front. Microbiol. 8:1396. 10.3389/fmicb.2017.01396 28790993 PMC5522875

[ref45] Sheng L. Hanrahan I. Sun X. Taylor M. H. Mendoza M. Zhu M.-J. (2018). Survival of *Listeria innocua* on Fuji apples under commercial cold storage with or without low dose continuous ozone gaseous. Food Microbiol. 76, 21–28. 10.1016/j.fm.2018.04.006, PMID: 30166144

[ref46] Smilanick J. L. Mansour M. Sorenson D. (2014). Performance of fogged disinfectants to inactivate conidia of *Penicillium digitatum* within citrus degreening rooms. Postharvest Biol. Technol. 91, 134–140. 10.1016/j.postharvbio.2013.12.020

[ref47] Suslow T. (2005). Chlorination in the production and postharvest handling of fresh fruits and vegetables. Available at: https://www.siphidaho.org/env/pdf/Chlorination_of_fruits_and_veggies.PDF (Accessed August 10, 2019).

[ref48] Torlak E. (2014). Inactivation of *Alicyclobacillus acidoterrestris* spores in aqueous suspension and on apples by neutral electrolyzed water. Int. J. Food Microbiol. 185, 69–72. 10.1016/j.ijfoodmicro.2014.05.022, PMID: 24929685

[ref49] Ukuku D. O. Fett W. F. (2006). Effects of cell surface charge and hydrophobicity on attachment of 16 *Salmonella* serovars to cantaloupe rind and decontamination with sanitizers. J. Food Prot. 69, 1835–1843. 10.4315/0362-028X-69.8.1835, PMID: 16924907

[ref50] Upadhyay A. Upadhyaya I. Mooyottu S. Kollanoor-Johny A. Venkitanarayanan K. (2014). Efficacy of plant-derived compounds combined with hydrogen peroxide as antimicrobial wash and coating treatment for reducing *Listeria monocytogenes* on cantaloupes. Food Microbiol. 44, 47–53. 10.1016/j.fm.2014.05.005, PMID: 25084644

[ref51] Yang X. J. Xu X. M. Xu J. Han Y. F. (2013). Iron oxychloride (FeOCl): an efficient Fenton-like catalyst for producing hydroxyl radicals in degradation of organic contaminants. J. Am. Chem. Soc. 135, 16058–16061. 10.1021/ja409130c, PMID: 24124647

[ref52] Yu T. T. Li Q. Zhao X. Y. Xia H. Ma L. Q. Wang J. L. (2017). Nanoconfined iron oxychloride material as a high-performance cathode for rechargeable chloride ion batteries. ACS Energy Lett. 2, 2341–2348. 10.1021/acsenergylett.7b00699

